# The Influence of Diabetes Mellitus and Kidney Dysfunction on Oxidative Stress, a Reflection of the Multisystem Interactions in Aortic Stenosis

**DOI:** 10.3390/antiox14070888

**Published:** 2025-07-18

**Authors:** Laura Mourino-Alvarez, Inés Perales-Sánchez, Germán Hernández-Fernández, Gabriel Blanco-López, Emilio Blanco-López, Rocío Eiros, Cristian Herrera-Flores, Miryam González-Cebrian, Teresa Tejerina, Jesús Piqueras-Flores, Pedro Luis Sánchez, Luis F. López-Almodóvar, Luis R. Padial, Maria G. Barderas

**Affiliations:** 1 Department of Vascular Physiopathology, Hospital Nacional de Parapléjicos, SESCAM, 45071 Toledo, Spain; lmourino@sescam.jccm.es (L.M.-A.); inesperalessanchez@gmail.com (I.P.-S.); ghernandezf@externas.sescam.jccm.es (G.H.-F.); gblancolopez99@gmail.com (G.B.-L.); 2 Department of Vascular Physiopathology, Hospital Nacional de Parapléjicos, IDISCAM, 45071 Toledo, Spain; emilioblanco96@gmail.com; 3 Department of Cardiology, Ciudad Real General University Hospital, 13005 Ciudad Real, Spain; 4 Department of Cardiology, Hospital Universitario de Salamanca, Instituto de Investigación Biomédica de Salamanca, Centro de Investigación Biomédica en Red de Enfermedades Cardiovasculares (CIBER-CV), 37007 Salamanca, Spain; reirosb@saludcastillayleon.es (R.E.); cristianherreracardio@gmail.com (C.H.-F.); miryamgcebrian@saludcastillayleon.es (M.G.-C.); pedrolsanchez@usal.es (P.L.S.); 5 Department of Pharmacology, School of Medicine, Universidad Complutense de Madrid, 28040 Madrid, Spain; teje@med.ucm.es; 6 Heart Failure and Inherited Cardiovascular Diseases Unit, Ciudad Real General University Hospital, 13005 Ciudad Real, Spain; jesus.piqueras.flores@gmail.com; 7 Faculty of Medicine, University of Castilla-La Mancha, 13071 Ciudad Real, Spain; 8 Cardiac Surgery, Hospital General Universitario de Toledo, SESCAM, 45007 Toledo, Spain; lopezalmodovar@yahoo.es; 9 Department of Cardiology, Hospital General Universitario de Toledo, SESCAM, 45007 Toledo, Spain; lrodriguez@sescam.org

**Keywords:** aortic stenosis, kidney dysfunction, diabetes mellitus, oxidative stress, thiols, albumin

## Abstract

Progression of aortic stenosis (AS) is aggravated by type 2 Diabetes Mellitus (T2DM) and kidney dysfunction (KD). Oxidative stress is one of the main mechanisms that triggers AS and is also disturbed among subjects with T2DM and KD. Consequently, we studied the redox homeostasis in four groups of patients, also classifying each patient based on their kidney function: control subjects, T2DM, AS, and AS+T2DM. Free reduced thiols in plasma were analyzed using a colorimetric assay, and the redox state of human serum albumin (HSA) was assessed by immunodetection and PEG-PCMal labeling. Lower levels of thiols were evident in patients with AS and AS+T2DM, while reduced and mildly oxidized HSA was more abundant in T2DM and AS+T2DM patients, reflecting less protection against oxidation. Moreover, the thiol levels decreased as KD increased in patients with AS and AS+T2DM. Differences also exist in reduced and mildly oxidized HSA between patients with normal and severely impaired kidney function, whereas AS patients with severe KD had more strongly oxidized HSA. Our results confirm an imbalance in oxidative stress associated with AS that is aggravated by the coexistence of T2DM and KD. Moreover, T2DM treatment might mitigate this dysfunction, opening the door to new therapeutic approaches for these patients.

## 1. Introduction

Aortic stenosis (AS) is the most common acquired heart valve disease, with an increasing prevalence due to the aging population. It is characterized by a progressive deterioration and thickening of the aortic valve (AV), preventing the valve from opening properly and restricting blood flow from the left ventricle to the rest of the body [1,2]. AS is usually diagnosed in a severe stage due to its long asymptomatic phase, yet once diagnosed, its progression is fast, associated with a poor prognosis and with only one therapeutic option: AV replacement [3].

Type 2 Diabetes Mellitus (T2DM) is a chronic disease characterized by chronic hyperglycemia that is a well-established cardiovascular risk factor. This metabolic disorder is related to AS since it has been reported to be a risk factor for AS progression [4]. Indeed, an increased prevalence of T2DM has been demonstrated in patients with AS and there is a higher incidence of AS among individuals with diabetes [5,6]. Several mechanisms might underlie this relationship, including inflammation, oxidative stress, and calcification, although the dynamics of these associations are not fully understood [4,7,8]. These complex interactions are also affected by kidney dysfunction (KD), which is closely associated with T2DM and that has also been proposed to be an important pathophysiological event in AS due to the associated mineral and hemodynamic disruptions [9,10]. The coexistence of KD with T2DM or AS negatively affects patient prognosis, increasing morbidity and mortality [9,11]. It was recently proposed that the coincidence of different modifiable risk factors, including T2DM and KD, contribute to the increased risk of developing AS [12], which reinforces the need to study these pathologies holistically.

Oxidative stress appears to be more intense in patients with AS and it has been suggested to be one of the mechanisms that triggers this pathology [13,14]. The increase in oxidative markers in patients with AS is coupled with an impaired antioxidant response [15]. Moreover, these mechanisms are also altered in patients with T2DM, which is associated with a pro-oxidant state [16] and defects in the antioxidant systems [17]. Similarly, oxidative stress is also associated with cardiovascular complications in patients with advanced chronic KD and with a faster progression of this disease [18]. Extracellular free thiols are reliable indicators of systemic redox status. These organosulfur compounds contain a free sulfhydryl (R–SH) group that is rapidly oxidized by reactive species, including reactive oxygen species (ROS), reducing the free thiol levels under conditions of oxidative stress [19]. Thiols are very abundant in the extracellular environment and they are considered one of the most important protective buffers against oxidative stress. Hence, oxidative stress can be easily monitored by measuring the levels of reduced thiols in plasma [20,21]. Importantly, human serum albumin (HSA) plays an essential role in maintaining redox hemostasis as it contains the largest amount of redox-active thiol groups [22]. The link between the redox state of albumin and different health conditions has been underlined repeatedly, even in AS, T2DM, and kidney disease [23]. Indeed, it is of note that the more intense expression of inflammatory cytokines by endothelial and valve interstitial cells cultured with oxidized albumin is associated with accelerated calcification [24].

In this study, we evaluated the oxidative stress in patients with isolated AS, T2DM and KD, comorbid AS-T2DM, and comorbid AS-T2DM-KD by measuring circulating free thiols, as well as by studying the redox state of HSA in plasma samples. Our results demonstrate the harmful effect of comorbidities in AS patients, paving the way for new therapeutic options for these patients and highlighting the unique pathophysiology of the comorbid state. This kind of study is essential to advance towards precision medicine, suggesting new avenues for research and for the development of new strategies to delay the progression of AS, optimizing patient management.

## 2. Materials and Methods

### 2.1. Patient Selection

Patients with severe AS, with and without T2DM, were recruited to this study at the Cardiac Surgery unit of the Hospital Universitario de Toledo (Toledo, Spain), while patients without AS were enrolled at the Hospital Virgen del Valle (Toledo, Spain). The participants were assigned to one of the four study groups based on their medical records: (i) control subjects, without T2DM or AS and 2 or fewer cardiovascular risk factors; (ii) patients with DM; (iii) patients with AS; and (iv) patients with AS and T2DM. In addition, the patients were also classified according to their kidney function, defined through the estimated glomerular filtration rate (eGFR) and defined by the criteria of the National Kidney Foundation as follows: ≥60, normal or mild loss of kidney function (eGFRnor); 45–59, mild to moderate loss of kidney function (eGFRmod); and ≤44, moderate to severe loss of kidney function (eGFRsev). Patients with any severe morbidity (ischemic heart disease with ventricular dysfunction, end-stage chronic kidney disease, dialysis), bicuspid AV, or a family or personal history of aortopathy, rheumatic valve disease, or moderate and severe mitral valve disease were excluded from the study.

Blood samples were collected from patients who underwent AV replacement in tubes containing EDTA, centrifuged at 1125× *g* for 15 min, and the resulting supernatant was immediately frozen at −80 °C for later analysis.

This study was carried out in accordance with the recommendations of the Helsinki Declaration and it was approved by the Ethics Committee at the Hospital Universitario de Toledo (CEIC 878, 29 June 2022). Signed informed consent was obtained from all subjects prior to their inclusion on the study.

### 2.2. Serum Free Thiol Levels

Thiol compounds in plasma samples were assayed as described previously [25], using a SensoLyte^®^ Thiol Quantitation Assay Kit (AnaSpec, Fremont, CA, USA) according to the manufacturer’s instructions. This assay is based on the reaction of the thiols’ sulfhydryl groups with Ellman′s Reagent, generating 2-nitro-5-thiobenzoic acid (TNB) that produces a yellow color that can be detected at 420 nm absorbance. The intensity of the color produced is proportional to the thiol concentration and it is expressed as µM thiol.

### 2.3. Immunodetection

To analyze albumin oxidation, samples were first labeled with SulfoBiotics PEG-PCMal (SB20-01, Dojindo Molecular Technologies Inc., Rockville, MD, USA), a 5 kDa Protein-SHifter, in accordance with the manufacturer’s instructions and as described previously [26]. Briefly, samples were incubated with PEG-PCMal at 37 °C for 30 min prior to their separation by electrophoresis, resolving equal amounts of protein (5 µg) from each patient on 8% SDS–PAGE gels in a Bio-Rad Miniprotean II electrophoresis cell run at a constant current of 35 mA/gel (Hercules, CA, USA). The gels were then exposed to UV light on a transilluminator to remove the Protein-Shifter prior to their transfer to a nitrocellulose membrane for 40 min at a constant voltage of 20 V. The membranes were then blocked for 1 h with 7.5% non-fat dry milk diluted in phosphate-buffered saline containing 0.5% Tween 20 (PBS-T) and probed overnight with a mouse monoclonal antibody against HSA (Abcam, Cambridge, UK, ab10241) diluted in 1/1000 PBS-T with 5% non-fat dry milk. After washing, the membranes were incubated with a specific HRP-conjugated anti-mouse secondary antibody (Cell Signaling, Danvers, MA, USA, #7076P2) diluted 1/1000 in PBS-T containing 5% non-fat dry milk and antibody binding was detected by enhanced chemiluminescence (ECL: GE Healthcare, Chicago, IL, USA), according to the manufacturers’ instructions. Densitometry was performed with the ImageQuantTL software v8.1 (GE Healthcare, Chicago, IL, USA) and the number of reduced cysteine (Cys) residues was determined based on the molecular mass shift of the bands after electrophoresis.

### 2.4. Statistical Analysis

Statistical analyses were performed using R software (RStudio 2024.12.0+467). The Kruskal–Wallis ANOVA with Dunn post hoc analysis was used for multiple comparisons of continuous variables, whereas the chi-square test was applied for categorical variables. Spearman correlation coefficients were calculated for the correlation analysis. The descriptive data are presented as the means ± standard deviation (SD) or as percentages. Statistical significance was accepted when the *p*-value < 0.05.

## 3. Results

In this study, we analyzed clinical samples to determine the oxidative status of four groups of subjects: controls without AS or T2DM, subjects with T2DM alone, patients with AS alone, or patients with both AS and T2DM. These subjects were also separated according to their renal function: eGFRnor, eGFRmod, or eGFRsev. Plasma thiol levels were measured, and the redox state of HSA was investigated in order to determine whether T2DM and renal function influence the redox state in AS.

### 3.1. Characteristics of the Study Population

The study population included 48 subjects (35% female), with a mean age of 78.5. There were no significant differences between the study groups regarding age, gender distribution, or prevalence of the cardiovascular risk factors, with the exception of T2DM and glycemia (Table 1). In order to minimize the presence of confounding factors, all the subjects recruited presented with arterial hypertension (AHT), a risk factor that could not be avoided due to the high prevalence of AHT in elderly people with AS.

### 3.2. Plasma Thiol Levels and Albumin Redox State

**Figure 1:**
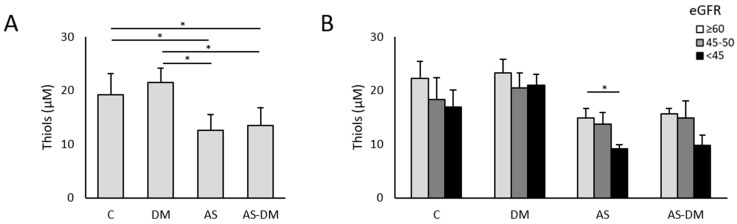
Free reduced thiol levels in plasma samples. (**A**) Levels of free reduced thiols in plasma from the 4 study groups. (**B**) Levels of free reduced thiols in plasma according to the eGFR. * *p*-value < 0.05.

After measuring the thiols in plasma, lower levels of reduced thiols were evident in both groups of patients with AS (AS 12.53 ± 3.02 µM; AS+T2DM 13.46 ± 3.34 µM) relative to both groups of patients without AS (C 19.13 ± 3.94 µM; T2DM 21.53 ± 2.58 µM: Figure 1A). In all these comparisons we found significant differences, with an adjusted *p*-value < 0.05 (Table 2).

**Figure 2:**
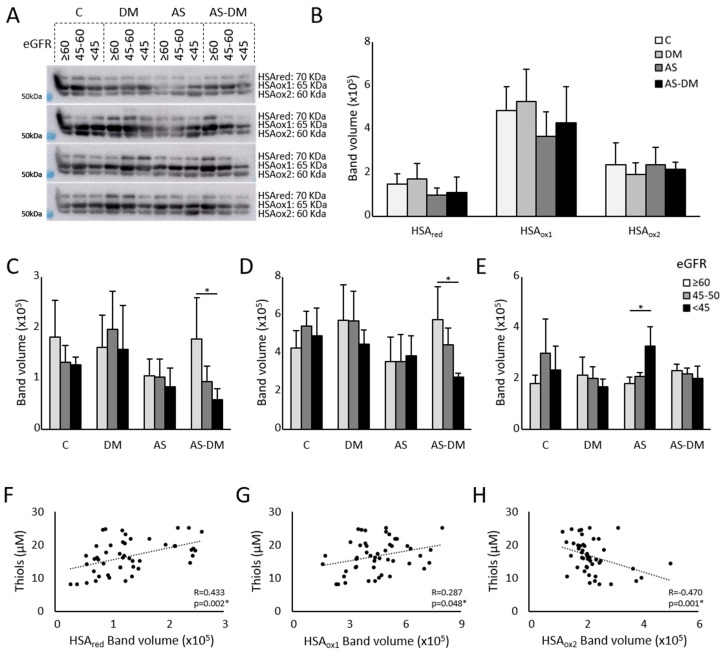
The redox state of human serum albumin in plasma samples. (**A**) Western blot of HSA labeled with the Sulfobiotics reagent detected in three different redox states. (**B**) Relative quantification of the three redox states of albumin: HSAred, HSAox1, and HSAox2. (**C**–**E**) The results expressed according to the eGFR of HSAred (**C**), HSAox1 (**D**), and HSAox2 (**E**). (**F**–**H**) Correlation between the thiol levels and the different HSA redox states: HSAred (**F**), HSAox1 (**G**), and HSAox2 (**H**). * *p*-value < 0.05.

Through PEG-PCMal labeling, we were able to visualize changes to the redox state of circulating HSA by electrophoretic analysis and immunodetection. There were three bands that corresponded to HSA, each separated by about 5 KDa: a more reduced basal state (HSAred); the basal state + 1 oxidation (HSAox1); and the basal state + 2 oxidations (HSAox2: Figure 2A). ANOVA analysis of the data from the immunoblots highlighted differences between the groups in terms of HSAred (*p*-value = 0.019) and the HSAox1 isoforms (*p*-value = 0.046), which accumulated more strongly in healthy subjects and patients with T2DM (Figure 2B, Table 2). After the ANOVA analysis, we did not find any differences between the study groups in terms of the most oxidized form of HSA, HSAox2 (*p*-value = 0.146).

### 3.3. The Relationship Between Free Reduced Thiols and Albumin Oxidation

In order to determine if an association exists between the free reduced thiols in plasma and the circulating HSA redox state, we calculated the correlation coefficient between the thiol levels and each HSA state: HSAred, HSAox1, and HSAox2 (Figure 2F–H). The correlation coefficient indicated there was a significant relationship between the thiol levels and the HSA redox state: HSAred (*p*-value = 0.002), HSA1ox (*p*-value = 0.048), and HSAox1 (*p*-value = 0.001). While the thiol levels were positively correlated with HSAred (ρ = 0.433) and HSAox1 (ρ = 0.287), they were negatively correlated with HSAox2 (ρ = −0.470), such that higher levels of reduced thiols were associated with lower levels of HSAox2 and vice versa.

### 3.4. Relationship Between Kidney Function, Free Thiols, and the Albumin Redox State

**Figure 3:**
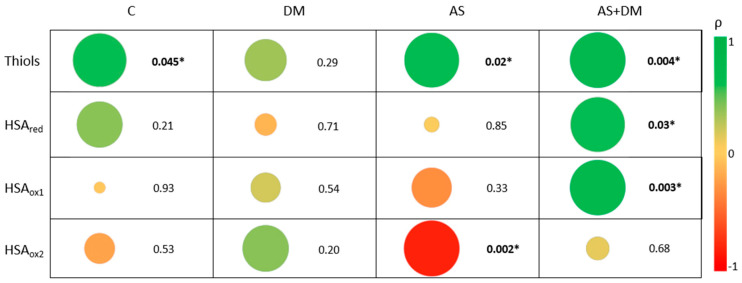
Bubble chart of the correlations between the parameters studied (thiols, HSAred, HSAox1, and HSAox2, and eGFR) in the different patient groups: controls (C), patients with DM (DM), patients with AS (AS), and patients with AS and DM (AS+DM). The area is proportional to the *p*-value, which is also shown, while the color is indicative of the R2 coefficient. * *p*-value < 0.05.

Having evaluated the differences between the four study groups, we set out to define if these relationships were affected by altered kidney function (Supplementary Table S1 and Supplementary Table S2). We first found a decrease in the free thiol levels in patients with impaired kidney function, especially in those with AS and AS+T2DM (Figure 1B). The amounts of reduced thiols in AS patients differed between those with the most severe kidney damage (eGFRsev = 9.08 ± 0.76 µM) and those with normal–moderately impaired kidney function (eGFRmod = 13.70 ± 2.22 µM, *p*-value = 0.072; eGFRnor = 14.82 ± 1.82 µM, *p*-value = 0.042). Although it does not show statistical significance, the same trend was observed in patients with AS+T2DM, with lower thiol levels in the eGFRsev group (thiols = 9.38 ± 1.80 µM) than in those with eGFRnor (thiols = 15.64 ± 0.99 µM, *p*-value > 0.05) and eGFRmod (thiols = 14.90 ± 3.15 µM, *p*-value > 0.05). In the controls, we also observed a similar decrease although these differences were not significant (*p*-value = 0.138). Surprisingly, we did not detect differences in the thiol levels among the patients with T2DM alone (*p*-value = 0.246). When the samples are grouped according to the eGFR, clear differences emerge between patients with and without aortic stenosis (AS), along with a noticeable decline in thiol levels when kidney function is compromised (Figure S1A). A significant positive correlation was also evident between the thiol levels and eGFR in patients with AS and patients with AS+T2DM (ρ = 0.669, *p*-value = 0.02 and ρ = 0.762, *p*-value = 0.004, respectively. Figure 3).

Regarding the albumin redox state, differences were evident between patients with AS+DM and normal kidney function and those AS+DM patients with severely altered kidney function (Figure 2C–E), both in terms of HSAred (eGFRnor = 177,412 ± 81,283; eGFRsev = 57,855 ± 22,201: *p*-value = 0.043) and HSAox1 (eGFRnor = 575,433 ± 172,985; eGFRsev = 273,979 ± 19,354: *p*-value = 0.013). Patients with moderate to severe kidney dysfunction exhibited decreased levels of HSAred when they also had AS, whereas those in the T2DM group showed higher levels (Figure S1B). In addition, patients with AS and a more severe deterioration in kidney function had higher levels of HSAox2 (eGFRsev = 326,256 ± 77,620) than patients with preserved kidney function (eGFRnor = 179,888 ± 25,408, *p*-value = 0.0013). In patients with AS+T2DM, the correlation analysis showed a significant increase in HSAred and HSAox1 when the eGFR was higher (ρ = 0.615, *p*-value = 0.03 and ρ = 0.783, *p*-value = 0.003, respectively). By contrast, in patients with AS, there was a significant negative correlation between HSAox2 and eGFR (Figure 3).

## 4. Discussion

A well-regulated redox state is vital to avoid physiological damage. Indeed, while ROS play a significant role in essential physiological functions, their overproduction may be harmful at a cellular and molecular level. This has been described in several pathologies, including AS, T2DM, and kidney disease [15,27,28,29,30]. The amount of free thiols in plasma is a reliable indicator of the systemic redox state of individuals as they are easily oxidized by ROS and other reactive species. As such, reduced free thiols act as antioxidants, neutralizing reactive species and thus protecting cells and tissue from the damage of oxidative stress, such that low levels of free thiols are suggestive of more intense oxidative stress [31]. Here, we measured the free reduced thiol levels in plasma from patients with AS and/or T2DM, classifying them according to their kidney function, enabling alterations to the redox state to be assessed when these conditions coexist. The global status of HSA was also evaluated as this is perhaps the most important source of circulating thiols.

As expected, we found lower thiol levels in patients with AS, irrespective of the presence of T2DM, indicating that patients with AS have a dampened antioxidant capacity. This is consistent with previous studies suggesting that AS alters the mechanisms involved in the activation of antioxidant defenses [15]. Thiol levels are correlated with the different redox states, although this correlation differs depending on the degree of albumin oxidation. Both HSAred and HSAox1 maintain an equilibrium with thiol levels, suggesting their implication in maintaining a redox hemostasis. However, the most oxidized form of albumin, HSAox2, is inversely related to the amount of thiols, indicating that when the levels of circulating thiols are diminished, a higher level of HSA oxidation is attained. HSA is the most abundant protein in plasma and it contains the highest number of redox-active thiol groups, contributing significantly to the antioxidant capacity of human plasma [22,32]. Although different sites of oxidation have been described [25,26,33], the free thiol group at Cyst-34 remains the best studied, and based on the oxidation of this site, three different fractions of HSA can be distinguished as follows: (1) the reduced form, with Cys34 as a thiol; (2) the mildly oxidized form, with Cys34 as a disulfide and with a low-molecular-mass thiol; and (3) a strongly oxidized form in which a sulfinic or sulfonic acid (di/trioxidation) appears [22,34]. While conversion between the reduced and the mildly oxidized form is reversible, the shift in HSA to a strongly oxidized form is thought to be permanent and reflects the status of different diseases like diabetes and kidney disease [27,35,36]. Although it has been proposed as a potential biomarker for the progression of kidney disease in patients with T2DM [37], here, we found that subjects without AS have higher levels of reduced and mildly oxidized HSA (HSAred and HSAox1), indicating they may respond better to an oxidative insult. In addition, patients with T2DM have slightly higher amounts of thiols, HSAred, and HSAox1, reinforcing the idea that pharmacological treatment for T2DM may improve the antioxidant response.

**Figure 4:**
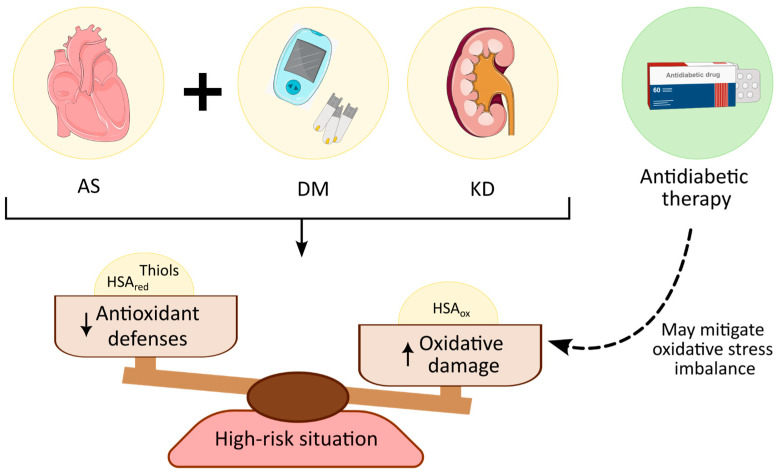
Summary of the main findings and conclusions of this work.

To evaluate the effect of KD in these patients, they were classified through their eGFR, showing that the most pronounced differences appear in patients with severe KD. Although some of these differences are not statistically significant, the potential biological meaning of these results should not be undervalued. In terms of the antioxidant response, a striking reduction in thiols exists in patients with AS and severe KD, as well as in healthy subjects, although these latter differences were more moderate. However, no such differences exist in patients with T2DM alone. Moreover, a progressive reduction in both HSAred and HSAox1 is observed, particularly in those with lower eGFR values, in patients with AS and T2DM, underscoring the heightened risk associated with the comorbid condition. Oxidative stress is a key factor in kidney pathologies and low levels of thiols are associated with less risk of KD [27,38]. Hence, the hypoglycemic drugs used to counteract oxidative stress [39,40] are likely to be less effective in patients with a low eGFR, especially if they also develop AS. In this situation, there seems to be a dysregulation of the albumin–thiol system and consequently, a more limited antioxidant capacity. Nevertheless, high levels of HSAox2 are only observed in patients with AS and severe KD and not in patients that also have T2DM. This was unexpected as previous studies into T2DM indicate that the albumin redox state shifts towards more oxidized fractions, reflecting disease status, and even constituting a prognostic indicator for disease progression and complications [35]. As a result, it is likely that antidiabetic treatments affect these levels of oxidation, reducing the irreversible damage produced by oxidative stress (Figure 4).

This study has several limitations, the most significant of which is the sample size that may limit the statistical power. To enhance the robustness of the statistical analysis in this situation, non-parametric tests have been used, although sensitivity is compromised. Moreover, the presence of comorbidities is a relevant concern. All the patients included in this study are hypertensive and although there were no differences between the study groups, some participants also present dyslipidemia and obesity. While group matching helps minimize the impact of these factors, their potential influence must still be borne in mind. As such, studies on larger cohorts will be necessary to support the results of this preliminary work and to delve deeper into the effect of hypoglycemic drugs in this kind of patient. In fact, the use of hypoglycemic drugs in AS patients undergoing TAVI has significantly reduced the incidence of death from any cause or worsening of heart failure [41], highlighting the importance of these studies.

## 5. Conclusions

Undoubtedly, we can conclude that the coexistence of AS, T2DM, and KD is a high-risk situation that compromises patient progression to an extreme. Compromising antioxidant defenses may be a crucial element in the progression of patients with AS and comorbidities like T2DM and KD. The role of antidiabetic therapy should also be studied in more depth, as it may delay the progression of AS and KD, which would represent an important improvement in the quality of life of these patients. This study highlights the convenience of studying these multifactorial pathologies as a whole, as they are tightly connected. This will be the only way to move forward towards preventive medicine, the cornerstone of precision medicine.

## Figures and Tables

**Table 1:** Clinical characteristics of the subjects studied: C, control subjects without calcific aortic valve disease or T2DM; T2DM, subjects with type 2 Diabetes Mellitus; AS, subjects with calcific aortic valve disease without type 2 Diabetes Mellitus; AS+T2DM, subjects with calcific aortic valve disease and type 2 Diabetes Mellitus. Abbreviations: AHT, arterial hypertension; eGFR, estimated glomerular filtration rate; HDL, high-density lipoprotein; LDL, low-density lipoprotein; M/F, male/female; TG, triglycerides.

Clinical Characteristics	C (n = 12)	T2DM (n = 12)	AS (n = 12)	AS+T2DM (n = 12)	*p*-Value
Age	79.7 ± 9.8	78.8 ± 8.2	79.5 ± 5.0	76.0 ± 4.1	0.273
Gender (M/F)	8/4	9/3	7/5	7/5	0.801
%AHT	100	100	100	100	-
%Dyslipidemia	42	83	83	67	0.090
%Diabetes	0	100	0	100	0.000
%Smokers	8.3	0	8.3	25	0.237
%Obesity	8.3	8.3	25	25	0.494
Cholesterol (mg/dL)	164.7 ± 60.1	163.1 ± 367	156.7 ± 41.5	135.6 ± 24.6	0.301
TG (mg/dL)	111.7 ± 52.9	139.8 ± 52.9	101.5 ± 37.0	101.1 ± 27.9	0.179
HDL (mg/dL)	51.06 ± 13.6	46.2 ± 16.8	46.8 ± 12.9	42.5 ± 14.2	0.334
LDL (mg/dL)	88.0 ± 45.8	88.8 ± 31.5	89.6 ± 30.0	74.2 ± 21.6	0.455
Glycemia (mg/dL)	96.2 ± 10.9	119.4 ± 34.0	97.1 ± 18.9	136.9 ± 54.7	0.030
eGFR	53.1 ± 17.7	54.4 ± 17.4	56.9 ± 17.7	49.3 ± 20.2	0.90
Creatinine (mg/dL)	1.34 ± 0.34	1.29 ± 0.39	1.16 ± 0.29	1.35 ± 0.55	0.47
Uric acid (mg/dL)	6.37 ± 2.00	6.04 ± 1.62	5.74 ± 1.32	6.69 ± 1.61	0.32

**Table 2:** Data from the comparisons between groups, including ANOVA analysis and *p*-values of the multiple comparisons after Bonferroni adjustment: SD, standard deviation; NA, not applicable. * *p*-value < 0.05.

	Group	Mean ± SD	*p*-Value	Adj. *p*-Value	
Thiol (µM)	Control	19.13 ± 3.94	**0.000** *	AS+T2DM vs. AS **AS vs. C** **AS vs. T2DM** **AS+T2DM vs. C** **AS+T2DM vs. DM** C vs. T2DM	1.000 **0.005** **0.000** **0.023** **0.000** 1.000
	T2DM	21.53 ± 2.58			
	AS	12.53 ± 3.02			
	AS+T2DM	13.46 ± 3.34			
HSA_red_ (Band volume)	Control	146.871 ± 49.361	**0.019** *	AS+T2DM vs. AS AS vs. C AS vs. T2DM AS+T2DM vs. C AS+T2DM vs. DM C vs. T2DM	1.000 0.186 0.086 0.284 0.138 1.000
	T2DM	171.749 ± 7.1120			
	AS	97.037 ± 33.570			
	AS+T2DM	109.596 ± 70.194			
HSA_ox1_ (Band volume)	Control	486.392 ± 10.9618	**0.046** *	AS+T2DM vs. AS AS vs. C AS vs. T2DM AS+T2DM vs. C AS+T2DM vs. DM C vs. T2DM	1.000 0.275 0.059 1.000 0.496 1.000
	T2DM	528.408 ± 14.6578			
	AS	366.031 ± 115.268			
	AS+T2DM	430.884 ± 164.350			
HSA_ox2_ (Band volume)	Control	237.165 ± 101.422	0.146	NA	
	T2DM	193.445 ± 51.137			
	AS	237.780 ± 79.305			
	AS+T2DM	215.880 ± 33.788			

## Data Availability

Data are contained within the article and Supplementary Materials.

## Supplementary Materials

The following supporting information can be downloaded at: https://www.mdpi.com/article/10.3390/antiox14070888/s1, Table S1: Statistical results showing comparisons between patients according to their kidney function. Table S2: Statistical results showing comparisons between patients according to their pathology. * *p*-value <0.05. Figure S1: Free reduced thiols levels (A) and relative quantification of the three redox states of the albumin: HSAred (B), HSAox1(C) and HSAox2 (D) in plasma samples to observe the differences of the different pathologies grouped by eGFR. * *p*-value < 0.05.
