# Supplementary material for: The Influence of Diabetes Mellitus and Kidney Dysfunction on Oxidative Stress, a Reflection of the Multisystem Interactions in Aortic Stenosis
Source: Antioxidants (Basel). 2025 Jul 18;14(7):888. doi: 10.3390/antiox14070888 (PMC12292387; doi:10.3390/antiox14070888)
Supplement: Supplementary file 1 [file antioxidants-14-00888-s001.zip › antioxidants-3747372-supplementary.pdf]

Article

The Influence of Diabetes Mellitus and Kidney Dysfunction on Oxidative Stress, a Reflection of the Multisystem Interactions in Aortic Stenosis

Laura Mourino-Alvarez [1,2], Inés Perales-Sánchez [1,2], Germán Hernández-Fernández [1,2], Gabriel Blanco-López [1,2], Emilio Blanco-López [2,3], Rocío Eiros [4], Cristian Herrera-Flores [4], Miryam González-Cebrian [4], Teresa Tejerina [5], Jesús Piqueras-Flores [6,7], Pedro Luis Sánchez [4], Luis F. López-Almodóvar [8], Luis R. Padial [9] and Maria G. Barderas [1,2,*,†]

Table S1. Statistical results showing comparisons between patients according to their kidney function. Significance values were calculated using Kruskal-Wallis and Dunn’s test. SD, standard deviation; NA, not applicable. * p-value < 0.05.

		eGFR	Mean±SD	p-value	Adj. P-value	
Thiol	C	≥60	22.17±3.24	0.138	NA	
		45-59	18.33±4.07			
		<45	16.90±3.17			
	T2DM	≥60	23.24±2.57	0.246	NA	
		45-59	20.41±2.79			
		<45	20.95±1.99			
	AS	≥60	14.82±1.82	0.024	≥60 vs 45-59	0.844
		45-59	13.70±2.22		≥60 vs <45	0.042*
		<45	9.08±0.76		45-59 vs <45	0.072
	AS-T2DM	≥60	15.64±0.99	0.058	NA	
		45-59	14.90±3.15			
		<45	9.83±1.80			
HSA_{red}	C	≥60	181733±71632	0.735	NA	
		45-59	131584±33884			
		<45	127298±14554			
	T2DM	≥60	161152±63857	0.694	NA	
		45-59	196577±75424			
		<45	157519±86823			
	AS	≥60	104968±33822	0.694	NA	
		45-59	102490±35926			
		<45	83655±36542			
	AS-T2DM	≥60	177412±81283	0.049	≥60 vs 45-59	0.842
		45-59	93522±30343		≥60 vs <45	0.043*
		<45	57855±22201		45-59 vs <45	0.509
HSA_{ox1}	C	≥60	424849±91833	0.276	NA	
		45-59	541994±77201			
		<45	492335±142992			
	T2DM	≥60	571216±186226	0.368	NA	
		45-59	568082±156768			
		<45	445929±76880			
	AS	≥60	354557±130772	0.944	NA	
		45-59	356452±141188			
		<45	387087±103897			
	AS-T2DM	≥60	575433±172985	0.015	≥60 vs 45-59	0.980
		45-59	443241±88621		≥60 vs <45	0.013*
		<45	273979±19354		45-59 vs <45	0.187
HSA_{ox2}	C	≥60	178841±34180	0.059	NA	
		45-59	298293±136072			
		<45	234361±92223			
	T2DM	≥60	213199±70866	0.437	NA	
		45-59	200995±43783			
		<45	166141±32543			
	AS	≥60	179888±25408	0.015	≥60 vs 45-59	0.980
		45-59	207198±15407		≥60 vs <45	0.013*
		<45	326256±77620		45-59 vs <45	0.187

	AS-T2DM	≥60	231173±23871	0.584	NA
		45-59	217384±23667		
		<45	199085±48633		

Table S2. Statistical results showing comparisons between patients according to their pathology. Significance values were calculated using Kruskal-Wallis and Dunn's test. SD, standard deviation; NA, not applicable. * p-value < 0.05.

	eGFR	**Group**	**Mean±SD**	**p-value**	**Adj. P-value**	
Thiol	≥60	C	22.17±3.24	0.010*	C vs T2DM<br>C vs AS<br>C vs AS-T2DM<br>T2DM vs AS<br>T2DM vs AS-T2DM<br>AS vs AS-T2DM	1.000<br>0.086<br>0.188<br>0.056<br>0.128<br>1.000
		T2DM	23.24±2.57			
		AS	14.82±1.82			
		AS-T2DM	15.64±0.99			
	45-59	C	18.33±4.07	0.058	NA	
		T2DM	20.41±2.79			
		AS	13.70±2.22			
		AS-T2DM	14.90±3.15			
	<45	C	16.90±3.17	**0.008***	C vs T2DM<br>C vs AS<br>C vs AS-T2DM<br>T2DM vs AS<br>T2DM vs AS-T2DM<br>AS vs AS-T2DM	1.000<br>0.187<br>0.379<br>**0.023***<br>0.056<br>1.000
		T2DM	20.95±1.99			
		AS	9.08±0.76			
		AS-T2DM	9.83±1.80			
HSA_{red}	≥60	C	181733±71632	0.292	NA	
		T2DM	161152±63857			
		AS	104968±33822			
		AS-T2DM	177412±81283			
	45-59	C	131584±33884	0.178	NA	
		T2DM	196577±75424			
		AS	102490±35926			
		AS-T2DM	93522±30343			
	<45	C	127298±14554	**0.050***	C vs T2DM<br>C vs AS<br>C vs AS-T2DM<br>T2DM vs AS<br>T2DM vs AS-T2DM<br>AS vs AS-T2DM	1.000<br>0.950<br>0.105<br>1.000<br>0.128<br>1.000
		T2DM	157519±86823			
		AS	83655±36542			
		AS-T2DM	57855±22201			
HSA_{ox1}	≥60	C	424849±91833	0.128	NA	
		T2DM	571216±186226			
		AS	354557±130772			
		AS-T2DM	575433±172985			
	45-59	C	541994±77201	0.106	NA	
		T2DM	568082±156768			

		AS	356452±141188			
		AS-T2DM	443241±88621			
	<45	C	492335±142992	0.084	NA	
		T2DM	445929±76880			
		AS	387087±103897			
		AS-T2DM	273979±19354			
HSA_{ox2}	≥60	C	178841±34180	0.147	NA	
		T2DM	213199±70866			
		AS	179888±25408			
		AS-T2DM	231173±23871			
	45-59	C	298293±136072	0.159	NA	
		T2DM	200995±43783			
		AS	207198±15407			
		AS-T2DM	217384±23667			
	<45	C	234361±92223	**0.036***	C vs T2 DM<br>C vs AS<br>C vs AS-T2DM<br>T2DM vs AS<br>T2DM vs AS-T2DM<br>AS vs AS-T2DM	1.000<br>0.713<br>1.000<br>**0.023***<br>1.000<br>0.448
		T2DM	166141±32543			
		AS	326256±77620			
		AS-T2DM	199085±48633			

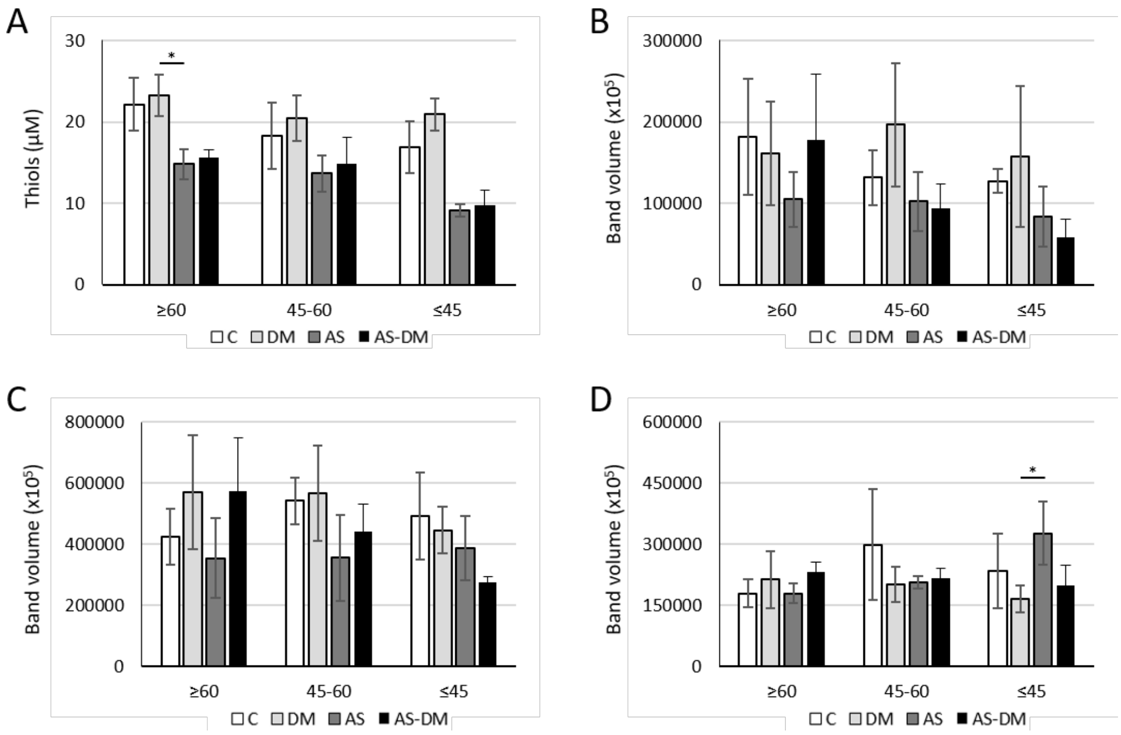

Figure S1. Free reduced thiols levels (A) and relative quantification of the three redox states of the albumin: HSAred (B), HSAox1(C) and HSAox2 (D) in plasma samples to observe the differences of the different pathologies grouped by eGFR. * p-value < 0.05.
